# Perioperative/postoperative anxiety and its interventions in gynecological cancers: a comprehensive review of clinical evidence

**DOI:** 10.3389/fpsyt.2024.1383029

**Published:** 2024-07-22

**Authors:** Beibei Chen, Chunyan Chen, Xiumin Zhao, Xiaoxia Wu

**Affiliations:** ^1^ Department of Gynecology, Hangzhou Women’s Hospital (Hangzhou Maternity and Child Health Care Hospital), Hangzhou, Zhejiang, China; ^2^ Department of Nephrology, Jiaxing Hospital of Traditional Chinese Medicine, Jiaxing, Zhejiang, China; ^3^ Department of Obstetrics and Gynecology, Tiantai Pingqiao Central Health Hospital, Tiantai People's Hospital of Zhejiang Province (Tiantai Branch of Zhejiang Provincial People's Hospital), Hangzhou Medical College, Taizhou, Zhejiang, China

**Keywords:** gynecological cancers, anxiety, perioperative, postoperative, nursing intervention

## Abstract

Gynecological cancers are prevalent malignancies among females, and surgical intervention is the primary therapeutic approach offering the possibility of a definitive cure. Recent research has highlighted the susceptibility of gynecological cancer patients to experiencing anxiety symptoms during the perioperative and postoperative phases, with this psychological condition being linked to suboptimal recovery following surgery. Nevertheless, certain interventions have shown promise in mitigating perioperative and postoperative anxiety in gynecological cancer patients. In this study, we conducted a comprehensive review to collect the existing evidence on this subject. Through a systematic search across six common databases, we screened and included 28 pertinent studies. The current review emphasizes the elevated occurrence of perioperative and postoperative anxiety among patients with gynecological cancers (i.e., uterine, cervical, ovarian, endometrial, and vulval cancers). Specific nursing interventions (i.e., crisis intervention nursing, multidisciplinary collaborative continuous nursing, psychological nursing, comprehensive psychological nursing, reminiscence therapy involved care, cognitive behavioral stress management, hospital-family integrated continuation nursing, high-quality nursing care, relaxation-focused nursing program, and relaxation/counseling intervention) and psychotropic medications may serve as dependable approaches to mitigate perioperative and postoperative anxiety. This study represents a novel contribution to the literature by providing a characterization of perioperative and postoperative anxiety in the context of gynecological oncology. The findings underscore the significance of addressing perioperative and postoperative anxiety as a critical clinical concern for individuals with gynecological cancers, emphasizing the need for further research to develop effective interventions.

## Introduction

Gynecological cancers are the categories of malignancies that occur in the reproductive organs or genitals of female subjects, accounting for over 14% of all cancers in women worldwide (1). It is reported that 40% of patients with gynecological cancers are premenopausal at the time of diagnosis (2, 3). The main types of gynecological cancer are cervical, ovarian, uterine, vaginal, and vulval, and are named for their respective organs (4). It is important to know that each type of cancer has a distinct burden on survivors’ quality of life (QoL) due to its symptoms. For example, there is an 85% chance of recurrence in ovarian cancer (5), and only 30% of patients survive 5 years after being diagnosed (6). With an increasing elderly population, the incidence of gynecological cancers is expected to increase further in the coming decades. Gynecological cancers are treated with modalities such as surgery, chemotherapy, and radiation therapy, depending on where they have spread (7). It would be helpful to develop algorithms and decision support tools that would guide clinicians in choosing optimal screening, therapeutic, and follow-up paths for patients with gynecological cancer (8). However, as the knowledge of these malignancies continues to evolve clinically, biologically, and pathologically, there remains a challenge posed by the cancer’s biological complexity and prognostic variability (9). Gynecological cancer, along with its associated treatment, causes both physical and mental changes (i.e., psychosocial and sexual consequences), adversely affecting a patient’s QoL (10). Even though gynecological cancers have improved considerably in terms of systemic treatment (i.e., chemotherapy, immunotherapy, and chemoimmunotherapy), surgical intervention remains the only method that can be relied upon to cure them permanently. Through management strategies aimed at minimizing harm and maximizing survival rates, QoL can be improved during the survivorship stage. Studies have shown that minimally invasive surgery (i.e., laparoscopic and robot-assisted approaches) can be highly beneficial for gynecological cancer patients (11).

As reported, the experience of anxiety and fear is frequently observed among patients upon their admission to the hospital for surgical procedures (12). Consequently, the combination of physiological alterations and psychological responses inherent in surgical interventions and hospitalization can potentially jeopardize the well-being of patients (13). Moreover, individuals scheduled for oncologic surgery encounter an even more arduous circumstance, as malignancy itself engenders considerable distress (14). The deleterious impact of perioperative and postoperative anxiety on patients’ recuperation is widely acknowledged, thereby potentially leading to adverse outcomes following surgery (15). Due to most of the gynecological cancer patients worrying about their illnesses, it was found that over 70% of the patients had higher anxiety and depression scores than healthy controls (16). In the perioperative and postoperative periods, the increased anxiety of the sufferers may be correlated to anesthetic use, greater pain after surgery, and prolonged hospital stays (17). Psychological issues have traditionally been underemphasized during clinical practice by clinicians and nurses.

Hence, it is imperative to effectively address anxiety symptoms and negative emotional responses to enhance the recovery process and overall well-being of individuals diagnosed with gynecological cancers. Psychotherapeutic interventions have shown promise in mitigating emotional distress among gynecological cancer patients (18), underscoring the need for increased focus on the psychosocial aspects of patient care, particularly during the perioperative and postoperative periods. Numerous studies have substantiated the efficacy of some specific interventions in this context, highlighting their potential to effectively assist gynecological cancer patients in managing perioperative and postoperative anxiety (19). We conducted the first comprehensive review of perioperative and postoperative anxiety in patients with gynecological cancer and its treatment in this study.

## Literature search and eligible study characteristic

**Figure 1:**
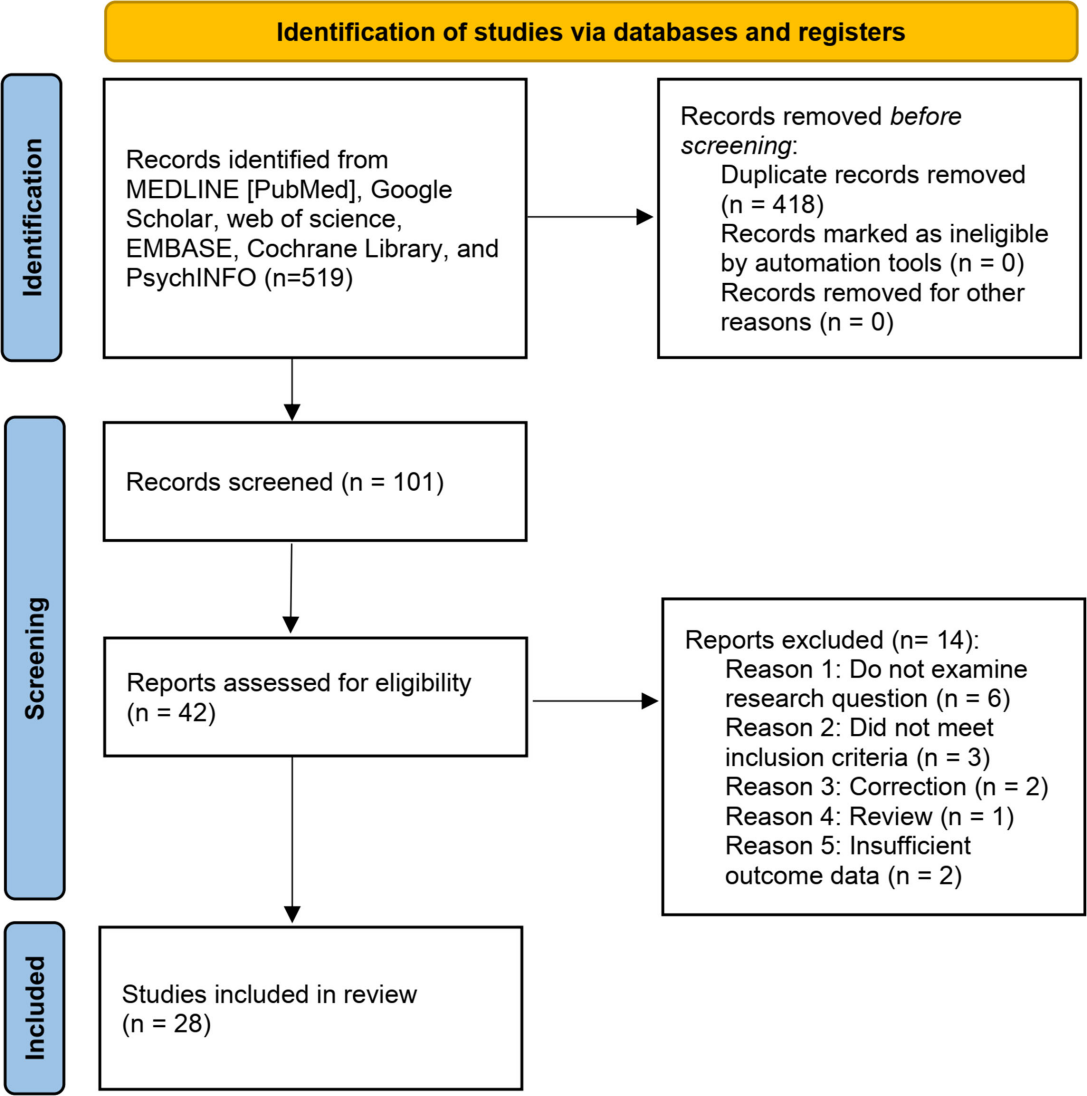
The PRISMA flow diagram.

We searched six general electronic databases (i.e., MEDLINE [PubMed], Google Scholar, Web of Science, EMBASE, Cochrane Library, and PsychINFO) to identify studies relevant to the topic of this review. Studies were retrieved up to January 1, 2024. We used the following screening strategies for screening the qualified publications in the MEDLINE database: (((((((Ovarian Cancer) OR (Uterine Cancer)) OR (Endometrial Cancer)) OR (Cervical Cancer)) OR (Vaginal Cancer)) OR (Vulvar Cancer)) AND (((((((((“Anxiety”[Mesh]) OR (Angst)) OR (Social Anxiety)) OR (Anxieties, Social)) OR (Anxiety, Social)) OR (Social Anxieties)) OR (Hypervigilance)) OR (Nervousness)) OR (Anxiousness))) AND (((perioperative) OR (postoperative)) OR (preoperative)). Additionally, the reference list of the relevant articles was manually examined to identify additional eligible studies. The exclusion criteria encompassed studies with duplicated data, reviews, letters, comments, meeting abstracts, case reports, and experimental experiments. The search process was independently conducted by two authors, with any uncertainties resolved by either a third author or the corresponding author. **Figure 1** illustrates the utilization of the PRISMA flow diagram to ascertain pertinent studies about perioperative and postoperative anxiety disorders in patients diagnosed with gynecological cancers. A total of 28 studies (15, 20–46) were deemed eligible for inclusion based on predetermined criteria. To facilitate the extraction of key data from these included studies, a standardized table for data collection was employed. These studies were published between 1993 and 2023 and were conducted in various geographical locations, including the USA, China, the UK, Italy, Sweden, Thailand, Australia, Japan, Canada, Turkey, and Korea. The sample sizes of the included studies ranged from 22 to 742 participants. The study designs encompassed randomized controlled trials (RCT), cohort, case-control, cross-sectional, prospective, retrospective study, and meta-analysis. The gynecological cancer types included uterine cancer (14 studies, **Table 1**), ovarian cancer (6 studies, **Table 2**), endometrial cancer (5 studies, **Table 2**), and other gynecologic and gynecologic cancers (1 study for cervix and vulva cancer studies and 2 studies without specific cancer type, **Table 2**). The evaluations for anxiety included State-Trait Anxiety Inventory (TAI), Hospital Anxiety and Depression Scale (HADS), Generalized Anxiety Disorder Scale (GAD), Depression and Anxiety Symptoms (IDAS), Self-rating anxiety scale (SAS), Patient-Reported Outcomes Measurement Information System (PROMIS), Hamilton anxiety scale (HAMA), the psychological well-being questions, quality of life questionnaire, and the Brief Profile of Mood States. In the following sections, we summarized and discussed the main findings from the 28 eligible studies.

**Table 1:** Perioperative/postoperative anxiety and its interventions in uterine cancer.

Study/Reference	Study design	Sample size	Cancer type/ intervention or management	Assessment for anxiety	Main findings
Caffo et al., 2003 (22) Italy	Prospective study	25 patients	Uterine cancer/postoperative radiotherapy	Diary card	Adjuvant pelvic radiotherapy after radical surgery for uterine cancer might result in anxiety disorder, which was more frequently observed in those with younger age (P<0.05).
Roberts et al., 2006 (23) UK	RCT	40 patients	Uterine cancer/radical surgery/intermittent self-catheterization	Quality of life questionnaire	As compared to suprapubic catheterization, intermittent self-catheterization was more acceptable for patients who underwent radical surgery for cervical cancer, allowing less anxiety/embarrassment and greater freedom to live a normal life.
Distefano et al., 2008 (24) Italy	Retrospective study	93 patients	Uterine cancer/chemoradiation or radical surgery	HADS	Locally advanced cervical cancer patients with administered preoperative chemoradiation had a significantly higher rate of anxiety than those who were at an early stage who underwent radical surgery (27.6% versus 8.6%, P=0.034).
Kobayashi et al., 2009 (26) Japan	Prospective study	60 patients	Uterine cancer/radiotherapy, chemoradiotherapy, or postoperative radiotherapy	HADS	No significant differences were observed in the scores of anxiety among the three treatment groups. However, the scores of anxiety was significantly lower in the high self-esteem group than that of the low self-esteem group (P=0.008).
Brotto et al., 2013 (27) Canada	Pilot study	34 patients	Uterine cancer/RH or RT	BAI	The prevalence of anxiety was significantly lower in the radical hysterectomy (RH) group than the radical trachelectomy (RT) group at 1 month after surgery (mean BAI score: 7.29 vs 7.75, P=0.005). However, the trend was reversed at 6 months after surgery (mean BAI score in RH and RT: 7.33 vs 2.55, P=0.032).
Wallin et al., 2019 (34) Sweden	Prospective study	26 patients	Uterine cancer/robot-assisted radical hysterectomy	The psychological well-being questions	Anxiety increased from baseline to 1 year after surgery in 17 of the 26 patients (65%). In addition, the patients also suffered from numbness of the labia, intercourse pain, lymphedema, and bladder problems.
Li et al., 2021 (37) China	Case-control study	151 patients	Uterine cancer/radical hysterectomy/crisis intervention nursing	HAMA	In patients with laparoscopic radical hysterectomy, those subjects underwent crisis intervention nursing (intervention countermeasures: cognition, psychology, behavior and society) had significantly lower HAMA scores than those who received conventional nursing measures.
Han et al., 2021 (36) China	Case-control study	132 patients	Uterine cancer/surgery/multidisciplinary collaborative continuous nursing	SAS	The anxiety scores in patients who received multidisciplinary collaborative continuous nursing (formulating a multidisciplinary collaborative continuous nursing plan) were significantly lower than those with regular continuous nursing.
Ma et al., 2021 (39) China	Case-control study	96 patients	Uterine cancer/surgery/traditional Chinese medicine for vaginal lavage combined with psychological nursing	SAS	As compared to the conventional nursing, anxiety was significantly reduced in those who underwent traditional Chinese medicine for vaginal lavage combined with psychological nursing. This combined method could also improve the patient’s immune function, T-lymphocyte subsets, and bone marrow hematopoietic function.
Shi et al., 2021 (40) China	Case-control study	109 patients	Uterine cancer/perioperative/comprehensive psychological intervention (ten days)	HADS	Cervical cancer patients who received a comprehensive psychological nursing intervention had lower scores of hospital anxiety and depression scale-anxiety than those who received conventional nursing intervention on day 10 after surgery. Also, the psychological nursing intervention also improved the patients’ negative emotions, social support degree, and immune functions.
Liu et al., 2021 (38) China	Case-control study	141 patients	Uterine cancer/postoperative chemotherapy/psychological nursing (3 months)	SAS	Patients who underwent postoperative chemotherapy with psychological nursing had a lower rate of anxiety than those with routine nursing at 30, 60, and 90 days of intervention (all P < 0.05). Furthermore, the SAS scale was negatively correlated with EROTC- Quality of Life Questionnaire-C30 scores (r= -0.4438, P < 0.05).
Liu et al., 2022 (42) China	RCT	152 patients	Uterine cancer/surgery/reminiscence therapy involved care (12 months)	HADS	Patients in the reminiscence therapy involved care (RTIC) group had a lower HADS-A score than those in the usual care group from 6 months to 12 months after intervention (all P < 0.05). RTIC could also reduce depression and improve the QoL in postoperative cervical cancer patients (all P < 0.05).
Yuan et al., 2023 (46) China	RCT	172 patients	Uterine cancer/postoperative/cognitive behavioral stress management (8 weeks)	SAS	Cognitive behavioral stress management dramatically reduced anxiety in patients with cervical cancer when compared to the patients who received normal care at 3 months (P=0.04) and 6 months (P=0.019) after discharge. This intervention also decreased depression and improved the QoL in patients with cervical cancer.
Li et al., 2023 (44) China	Case-control study	114 patients	Uterine cancer/postoperative/hospital-family integrated continuation nursing	The Brief Profile of Mood States	The anxiety scores in the hospital-family integrated continuation nursing group were significantly lower than those in the routine nursing care group (P = 0.017). This nursing intervention also reduced depression (P = 0.009), fatigue rating (P = 0.012), and anger (P <.001) in the postoperative uterine cancer patients.

STAI, State-Trait Anxiety Inventory; HADS, Hospital Anxiety and Depression Scale; BAI, BECK anxiety inventory; HAMA, The Hamilton Anxiety Scale; SAS, self-rating anxiety scale; QoL, Quality of life.

**Table 2:** Perioperative/postoperative anxiety and its interventions in ovarian, endometrial, and other gynecological cancers.

Study/Reference	Study design	Sample size	Cancer type/ intervention or management	Assessment for anxiety	Main findings
Ovarian cancer					
Schulman-Green et al., 2008 (25) USA	Retrospective study	145 patients	Ovarian cancer/postoperative	SAS	Patients who had greater symptom distress might have more anxious disorders. Furthermore, patients who were younger, more educated, and had early-stage disease had the lowest QoL.
Chittrakul et al., 2015 (29) Thailand	Cross-sectional study	56 patients	Ovarian cancer/surgery	HADS	The prevalence of anxiety was comparable between the ovarian cancer groups and the non-cancer group (at 7.1% each). Also, the HADS scores for anxiety were not significantly different between the cancer and non-cancer groups (5.0 vs 6.1, P=0.09).
Jang et al., 2017 (32) Korea	RCT	22 patients	Ovarian cancer/surgery/propranolol (40 mg two days before and 3 days after surgery)	STAI	The change of anxiety score (STAI) was not different between the propranolol group and the placebo group (-.6.1 vs -5.0, P=0.79). However, perioperative propranolol was found to be effective in reducing tumor burden.
Jin et al., 2022 (41) China	Meta-analysis	742 patients	Ovarian cancer/perioperative period/high-quality nursing care	SAS	As compared to routine nursing care, ovarian cancer patients who underwent high-quality nursing care had a more promising effect on anxiety relief (P <.001) and decreased depression (P<.001) during the perioperative period.
He et al., 2023 (15) China	Retrospective study	258 patients	Ovarian cancer/perioperative period	SAS	Patients were prone to anxiety during the perioperative period, which seriously affected the treatment effect. The stress factors for anxiety were the trauma of surgery and chemotherapy, pain, and the high treatment cost.
Güler et al., 2023 (43) Turkey	RCT	46 patients	Ovarian cancer/surgery/relaxation-focused nursing program	STAI	The relaxation-focused nursing program was more effective than conventional nursing care for preoperative anxiety, characterized by significantly reduced anxiety scores (P <.001).
Endometrial cancer					
Ferrandina et al., 2014 (28) Italy	Prospective study	132 patients	Endometrial cancer/surgery	HADS	The rate of anxiety disorders (score ≥11) in endometrial cancer patients after surgery was 19.5%, while this rate dropped to 12.3% at 3 months after surgery and to 6.2% at 24 months after surgery.
Honerlaw et al., 2016 (31) USA	Case-control study	71 patients	Endometrial cancer/recovering from surgery	IDAS	Patients experienced the most severe pain when anxiety was highest. Anxiety was one of the important risk factors for pain after surgery.
Sanjida et al. 2019 (33) Australia	RCT	719 patients	Endometrial cancer/surgery/psychotropic medications	NA	The prevalence of anxiety in patients with endometrial cancer was 2.5% (18/719). Only a few women received a new prescription of psychotropic medications after surgery.
Wang et al., 2020 (35) China	Retrospective study	714 patients	Endometrial cancer/undergoing surgery	HAMA	The incidence of postoperative anxiety in patients with endometrial cancer was 15.55%. Postoperative pain (OR=3.166, P<0.001) and combined liver disease (OR=2.318, P=0.001) were independent risk factors for anxiety disorder in patients after surgery.
Lindfors et al., 2023 (45) Sweden	Prospective study	64 patients	Endometrial cancer/robotic surgery	GAD-7	The incidence of anxiety in endometrial cancer patients before surgery was 27% (17/64), while it decreased to 7% (4/64) 2 weeks after robotic surgery (P=0.012). The preoperative anxiety symptoms reduced promptly after surgery, while the proportion of depression remained constant during the 1-year follow-up (P= 0.58).
Other gynecologic and gynecologic cancers					
Corney et al., 1992 (20) UK	Retrospective study	138 patients	Cervix and vulva cancers/surgery	HADS	Sexual problems after surgery were significantly associated with the level of anxiety of the patients.
Petersen et al., 2002 (21) Australia	RCT	50 patients	Gynaecological cancer/post-operative period/relaxation and counselling intervention (6 months)	HADS	Patients who received a relaxation and counselling session had significantly reduced anxiety subscale scores (P<0.02). The exercise included conscious breathing, body scan, progressive muscle relaxation, and guided imagery.
Doll et al., 2016 (30) USA	Prospective study	281 patients	Gynecologic and gynecologic cancers/undergoing surgery	PROMIS	As compared to the baseline score of anxiety, postoperative patients had a significantly increased probability of anxiety at 1 month after surgery (OR=2.5, 95%CI: 1.2–5.0). Anxiety was a lasting effect of temporary surgical complications.

STAI, State-Trait Anxiety Inventory; HADS, Hospital Anxiety and Depression Scale; BAI, BECK anxiety inventory; HAMA, The Hamilton Anxiety Scale; IDAS, Depression and Anxiety Symptoms; HAMA, Hamilton Anxiety Scale; PROMIS, Patient-Reported Outcomes Measurement Information System; SAS= self-rating anxiety scale; QoL, Quality of life; GAD, Generalized Anxiety Disorder Scale.

## Uterine cancer

### Postoperative radiotherapy, chemoradiation, and chemotherapy

Uterine cancer, the most common malignancy of the female genital tract, is one of the few cancers with elevating incidence and mortality, which is partially related to the high prevalence of obesity and overweight since the 1980s (47). In the surgical option of uterine cancer, laparotomy has been replaced by a more minimally invasive treatment, i.e., laparoscopic and robot-assisted hysterectomy (48). However, whichever the surgical procedure is, psychiatric disorders are the common complications in the perioperative and postoperative periods of uterine cancer patients. In this review, there were 14 included studies that reported the perioperative and postoperative anxiety of patients with uterine cancer. The prevalence of anxiety in these patients among different studies ranged from 8.6% to 65% (**Table 1**).

Radiation therapy administered after hysterectomy (cervix or corpus) is widely used for improving local control in patients with uterine cancer (49). However, adjuvant radiation therapy may induce acute side effects which can be a key source of emotional and physical distress of the sufferers. Furthermore, emotional disorders may further lead to poor treatment adjustment and a worsening quality of life. Therefore, clinicians should be aware of the detrimental psychological effects when performing postoperative pelvic radiation. Two included studies reported anxiety disorders in uterine cancer patients who received postoperative radiotherapy. Caffo et al. (22) recruited 25 uterine cancer patients who underwent postoperative radiotherapy and evaluated their anxiety symptoms by using the diary card. The authors found that adjuvant pelvic radiotherapy after radical surgery for uterine cancer might result in anxiety disorder and the levels of anxiety were stably low throughout the treatment period. In the subgroup analysis, anxiety was more frequently observed in those of a younger age in the second to fifth week of treatment (P<0.05). In patients with locally advanced cervical cancer (i.e., stages Ib2, IIb< 4 cm in diameter, IIIb, or IV), concomitant chemoradiotherapy was suggested. This study demonstrated that the levels of anxiety were low in patients who underwent adjuvant pelvic radiotherapy after radical surgery. Kobayashi et al. conducted a prospective study to compare the anxiety status of the patients who received radiotherapy, chemoradiotherapy, or postoperative radiotherapy. The results showed that no significant differences were observed in the scores of anxiety among the three treatment groups. Also, the anxiety scores were comparable between the early-stage and advanced-stage groups (P=0.866). However, the scores of anxiety were significantly lower in the high self-esteem group than that of the low self-esteem group, in any of the three treatment groups (all P<0.05). This study demonstrated that there were no significant differences among treatment modalities and disease stages in the context of prospective anxiety of uterine cancer patients. The impact of chemoradiation therapy on the quality of life in cervical cancer survivors has not been studied in-depth for a long time. Distefano et al. (24) found that locally advanced cervical cancer patients with administered preoperative chemoradiation had a significantly higher rate of anxiety than those at an early stage undergoing radical surgery (27.6% versus 8.6%, P=0.034). The high levels of HADS-anxiety might be correlated with the cancer stage, showing that patients with locally advanced cervical cancer were more vulnerable to anxiety than those with early-stage disease. Chemotherapy drugs have been found to dramatically affect patients’ physical functions and reduce their self-recognition due to significant side effects and long treatment cycles (50). Liu et al. (38) reported that long-term anxiety symptoms were found in cervical cancer patients undergoing postoperative chemotherapy. Taken together, the above studies revealed a higher susceptibility of cervical cancer patients to the adverse effects of anxiety from postoperative radiotherapy, chemoradiation, and chemotherapy. Given this, psychosocial interventions may provide clinical benefit to these patients.

### Nursing interventions

Numerous studies have indicated that appropriate nursing care can help cancer patients cope with anxiety during perioperative procedures (51). To alleviate perioperative and postoperative anxiety in patients with uterine cancer, several nursing interventions have emerged gradually over the past few years. There were eight included studies indicating that nursing interventions were effective treatments for reducing uterine cancer patients’ perioperative and postoperative anxiety. In patients with laparoscopic radical hysterectomy, Li et al. (37) demonstrated that the patients with laparoscopic radical hysterectomy who underwent crisis intervention nursing (intervention countermeasures: cognition, psychology, behavior, and society) had significantly lower HAMA scores compared to those who received conventional nursing measures. This study implied that crisis intervention nursing was conducive to relieving the anxiety of uterine cancer patients. In line with Li et al.’s study, Han et al. (36) also found that the anxiety scores in patients who received multidisciplinary collaborative continuous nursing were significantly lower than those with regular continuous nursing. The nursing strategies included a multidisciplinary collaborative continuous nursing plan, involving face-to-face consultation and lectures, WeChat/telephone follow-up, diet, drugs, pain relief, psychological intervention, self-care, recognition and processing of postoperative complications, daily activities, and social behaviors.

Clinical nursing interventions may be an effective way to control the anxious emotions of patients suffering from uterine cancer. Shi et al. (40) showed that cervical cancer patients who received a comprehensive psychological nursing intervention had lower scores of hospital anxiety and depression scale-anxiety than those who received a conventional nursing intervention on day 10 after surgery. Also, such a psychological nursing intervention improves the patient’s negative emotions, social support degree, and immune functions. A comprehensive cognitive intervention was composed of health education, behavioral intervention, emotional intervention, and social support intervention. Similarly, Liu et al. (38) reported that patients who underwent postoperative chemotherapy with psychological nursing had a lower rate of anxiety than those with routine nursing at 30, 60, and 90 days of intervention (all P < 0.05). Furthermore, the SAS scale was negatively correlated with EROTC- Quality of Life Questionnaire-C30 scores (r= -0.4438, P < 0.05). This study indicated that psychological intervention could reduce the patients’ short- and long-term anxious symptoms when they underwent postoperative chemotherapy. Reminiscence therapy involved care (RTIC) was combined with health education (i.e., introducing yourself, family, and hometown) and reminiscence therapy (i.e., sharing a story about school life, career, and hobbies).

Liu et al. (42) found that patients in the RTIC group had a lower HADS-A score than those in the usual care group from 6 months to 12 months after intervention (all P < 0.05). This RCT also demonstrated that RTIC could reduce depression and improve the QoL of postoperative cervical cancer patients (all P < 0.05). Cognitive behavioral stress management (CBSM) included disease-related education, pelvic floor rehabilitation training, stress management (i.e., narrating the physiological effects of stress, cognitive-behavioral explanations of stress and emotions, identifying cognitive disorders and automatic thinking, etc), and relaxation training. Yuan et al. (46) implied that CBSM dramatically reduced anxiety in patients with cervical cancer when compared to the patients who received normal care at 3 months (P=0.04) and 6 months (P=0.019) after discharge. This intervention also decreased depression and improved the QoL in patients with cervical cancer.

Integrated continuity nursing between hospitals and families is a new nursing concept that has recently been introduced as this intervention may provide continuous and coordinated nursing services after discharge. This nursing intervention has been proposed by a few cancer researchers (44). Li et al. reported that the scores of anxiety in the hospital-family integrated continuation nursing group were significantly lower than those in the routine nursing care group (P= 0.017) (44). This nursing intervention also reduced the depression (P= 0.009), fatigue rating (P= 0.012), and anger (P< 0.001) of postoperative uterine cancer patients (44). After cervical cancer surgery, patients who received hospital-family integrated continuation care were more likely to complete their treatment successfully than those who received conventional nursing care. The continuation of care was reported as a recovery of bad mood and improving the family function and sexual function of the patients. Chinese medicine has been used in the treatment of malignant tumors as well as cancer-related psychological disorders (52). As compared to conventional nursing, Ma et al. (39) reported that anxiety was significantly reduced in those who underwent traditional Chinese medicine for vaginal lavage combined with psychological nursing. This combined method could also improve the patient’s immune function, T-lymphocyte subsets, and bone marrow hematopoietic function. The above included studies revealed that psychological nursing intervention programs could effectively alleviate a patient’s anxious symptoms, indicating nursing interventions are essential for patients with uterine cancer during perioperative periods.

### Other specific interventions

Catheterization is one of the complementary means of early-stage uterine cancer following radical hysterectomy (53). Roberts et al. (23) performed an RCT to investigate the anxiety impact of different catheterizations in uterine cancer patients who received radical surgery. As compared to suprapubic catheterization, the authors found that intermittent self-catheterization was more acceptable for patients who underwent radical surgery for cervical cancer, allowing less anxiety/embarrassment and greater freedom to live a normal life. Women with early-stage cervical cancer wishing to preserve their fertility have increasingly chosen radical trachelectomy (RT), which leaves the uterus intact (54). In a pilot study, Brotto et al. (27) showed that the prevalence of anxiety was significantly lower in the radical hysterectomy (RH) group than in the RT group at 1 month after surgery (mean BAI score: 7.29 vs 7.75, P=0.005). However, the trend was reversed at 6 months after surgery (mean BAI score in RH and RT: 7.33 vs 2.55, P=0.032). The results of this study suggested that RT promoted positive emotional and sexual functioning and reduced sex-related distress significantly when compared to women receiving RH. Either open or laparoscopic radical hysterectomy for cervical cancer is associated with impaired sexual, bladder, and bowel functions (55, 56). Sexual, bladder, and bowel dysfunction are the common risk factors for the development of anxiety (57). Wallin et al. (34) reported that anxiety increased from baseline to 1 year after robot-assisted radical hysterectomy in 17 of 26 patients (65%). In addition, the patients also suffered from numbness of the labia, intercourse pain, lymphedema, and bladder problems. In this review, eight relevant studies reported the different nursing interventions for perioperative and postoperative anxiety in patients with uterine cancer. The characteristics of the aforementioned studies are displayed in **Table 1**.

## Ovarian cancer

There were expected to be approximately 22,240 new cases of ovarian cancer diagnosed and 14,070 deaths as a result of ovarian cancer in 2018 (58). Surgery is still the most effective method to cure ovarian cancer. Nevertheless, ovarian cancer patients are vulnerable to significant anxiety during the perioperative and postoperative periods. It is known that ovarian cancer patients are more vulnerable to psychological disorders such as anxiety (12). In a retrospective study (sample size: 145) developed by Green et al. (25), the authors found that patients who had greater symptom distress might have more anxiety disorders. Besides, patients who were younger, more educated, and had early-stage disease had the lowest QoL. Because young women have more to deal with (e.g., children, jobs) when it comes to integrating their health issues into their daily lives, they are more distressed. Women with higher levels of education may be better able to find and understand information about ovarian cancer, leading to more concern about the implications of their disease, the treatment, and the prognosis. Newly diagnosed women might be overwhelmed and frightened of the cancer, and thus have more mental disorders. Consistent with Green et al.’s findings, He et al. (15) also found that patients were prone to anxiety during the perioperative period, which seriously affected the treatment effect. The stress factors for anxiety were the trauma of surgery and chemotherapy, pain, and the high treatment cost.

In line with the effects of nursing interventions in uterine cancer-related perioperative anxiety, nursing intervention programs also worked equally well on ovarian cancer-associated anxiety disorder. Güler et al. (43) created a relaxation-focused nursing program for patients before ovarian cancer surgery. This program included initiating communication with the patients, encouraging the patient to express their feelings, creating a positive environment, providing information to alleviate stress and anxiety, performing relaxation exercises, and helping the patient relax. The results indicated that a relaxation-focused nursing program was more effective than conventional nursing care for preoperative anxiety, which was shown by significantly reduced anxiety scores (P < 0.001). Consistently, in a large-sample meta-analysis conducted by Jin et al. (41), the authors demonstrated that ovarian cancer patients who underwent high-quality nursing care had a more promising effect on anxiety relief (MD= −9.00, 95%CI: -11.36 to -6.63, P <.001) as well as depression decrease (MD= −7.62, 95%CI: −8.45 to −6.78, P<.001) than the routine nursing care during the perioperative period. High-quality nursing care mainly included psychological counseling and advice. The above included studies provided evidence of nursing interventions for anxiety disorder relief in patients with ovarian cancer during the perioperative period.

Cancer-related surgery is an important contributor to stress. Propranolol is a ß-blocker (inhibiting beta-adrenergic receptor signaling), which has been found to completely block the effects of surgical stress on cancer development (59). Jang et al. (32) investigated whether perioperative propranolol in patients with ovarian cancer undergoing surgery could reduce perioperative tumor growth mediated by surgical stress. The findings of this study showed that the change in anxiety score (STAI) was not different between the propranolol group and the placebo group (-.6.1 vs -5.0, P=0.79). However, though there was no significant benefit of propranolol on anxiety scores, perioperative propranolol was found to be effective in reducing the tumor burden. In a previous clinical study, Chittrakul et al. (29) reported that the prevalence of anxiety was comparable between the ovarian cancer groups and the non-cancer group (at 7.1% each). Also, the HADS scores for anxiety were not significantly different between the cancer and non-cancer groups (5.0 vs 6.1, P=0.09). According to the literature, baseline performance status and global quality of life assessments are predictive factors for progression-free survival (PFS) and overall survival (OS) in advanced ovarian cancer (60). In cases of advanced epithelial ovarian cancer, a poor performance status may warrant a more aggressive neoadjuvant chemotherapy approach (61). Patients with advanced ovarian cancer frequently exhibit poor performance status as a result of peritoneal or pleural carcinomatosis, potentially leading to heightened perioperative anxiety in individuals with gynecological malignancies. These studies implied that there was still controversy regarding the topic of anxiety development in the perioperative and postoperative periods of ovarian cancer (**Table 2**), indicating more relevant studies are still warranted to explore this scientific issue.

## Endometrial cancer

Unlike many other cancer types, endometrial cancer continues to increase in incidence (62). However, the 5-year relative survival rate of endometrial cancer is 84%, which is a relatively good prognosis (63). Nevertheless, endometrial cancer-related surgery also causes anxiety symptoms for the patients. Ferrandina et al. (28) reported that the rate of anxiety disorders (score ≥11) in endometrial cancer patients (sample size: 132) after surgery was 19.5%, while this rate dropped to 12.3% at 3 months after surgery and to 6.2% at 24 months after surgery. A previous case-control study (31) investigating patients recovering from surgery showed that patients experienced the most severe pain when anxiety was highest. This study indicated that anxiety was one of the important risk factors for pain after surgery. Wang et al. (35) conducted a large-sample retrospective study (714 participants) and demonstrated that the incidence of postoperative anxiety in patients with endometrial cancer was 15.55%. They further found that postoperative pain (OR=3.166, P<0.001) and combined liver disease (OR=2.318, P=0.001) were independent risk factors for anxiety disorders in patients after surgery. Robotic surgery is the most minimally invasive method for the treatment of endometrial cancer. Lindfors et al. (45) showed that the incidence of anxiety in endometrial cancer patients before robotic surgery was 27% (17/64), while it decreased to 7% (4/64) at 2 weeks after surgery (P=0.012). The preoperative anxiety symptoms reduced promptly after surgery, while the proportion of depression symptoms remained constant during the 1-year follow-up (P= 0.58). Since anxiety disorders are frequently observed in patients with endometrial cancer who underwent surgery, psychotropic medications may play a key role in alleviating anxiety in these patients. Sanjida et al. (33) found that the prevalence of anxiety in patients with endometrial cancer was 2.5% (18/719). However, only a few women received a new prescription of psychotropic medications after surgery. There were 16.8% of patients prescribed one or more psychotropic medications (n = 121/719). Antidepressants accounted for 12.6% of prescriptions, 91/719 were prescribed anxiolytics, and 42 were prescribed anxiolytics. This study revealed that overall prescription rates among endometrial cancer patients were higher than in the general population, but comparable to those among other cancer patients. Since women’s life expectancy has been increasing worldwide, age-related comorbidities (i.e., hypertension, diabetes mellitus, obesity, and cardiovascular disease) have received much attention as they may impair the achievement of treatment and influence the consequent prognosis of patients with gynecological cancers. Donato et al. assessed the impact of different comorbidities and concurrent medications used on the survival outcomes by using the age-adjusted Charlson comorbidity index score (A-CCI) in patients with endometrial cancer (64). A-CCI scores ≥3 have been found to be associated with more aggressive tumor features, higher risk of recurrence, and death due to disease. This study revealed that patients with endometrial cancer were featured by a high burden of comorbidities which are correlated directly to survival outcomes. In addition to comorbidities, age and concurrent medication use may also affect the survival of patients. This study indicated that A-CCI scores ≥3 might be an important risk factor for endometrial cancer patients who experience perioperative/postoperative anxiety. According to this fact, early interventions may benefit the patients. The summary of the above included studies reporting perioperative/postoperative anxiety in endometrial cancer patients is illustrated in **Table 2**.

## Other gynecologic and gynecologic cancers

In this review, three included studies reported anxiety disorders in the perioperative and postoperative periods of other gynecologic and gynecologic cancers. A previous retrospective study (sample size: 138 patients) reported that in cervix and vulva cancer patients, sexual problems after surgery were significantly associated with the level of anxiety of the patients (20). As compared to the baseline score of anxiety, Doll et al. (30) found that postoperative patients with gynecological cancer had a significantly increased probability of anxiety 1 month after surgery (sample size: 281, OR=2.5, 95%CI: 1.2–5.0). Anxiety was a lasting effect of temporary surgical complications. In an RCT developed by Rodney et al. in Australia (21), the investigators showed that patients who received a relaxation and counseling session intervention had significantly reduced anxiety subscale scores (P<0.02). The exercise included conscious breathing, body scan, progressive muscle relaxation, and guided imagery. As a result of the intervention, patients’ symptoms improved immediately, which made it a desirable endpoint in and of itself. Taken together, a review of studies on postoperative gynecological cancer patients indicates that anxiety is one of the psychological disorders in the patients, while nursing interventions can alleviate anxiety to some degree as well as improve quality of life (**Table 2**). At present, however, the evidence of nursing interventions for relieving anxiety in postoperative gynecological cancer patients is derived from limited studies. Therefore, more large-sample RCTs are still needed to validate the effects of nursing interventions.

## Other potential factors regarding anxiety in patients with gynecological cancers

The perioperative anxiety states of patients with gynecological cancers are influenced by a variety of factors that can have an impact preoperatively, intraoperatively, and postoperatively (37). These factors include specific surgery for different gynecological malignancies (i.e., cancer type, surgical type, risk level of surgery, preoperative preparation, and duration of surgery), personal characteristics (i.e., personality, emotional state, and social support), health status (i.e., disease severity and the understanding of the disease), pre-operative knowledge, education, psychological factors (self-perception, mental health status, and personal coping skills), and economic factors (i.e., medical costs, insurance coverage, and financial worries). All the aforementioned factors may induce personal anxiety and affect the efficiency of psychological interventions.

## Preventions of gynecologic malignancies

Prevention of gynecologic malignancies may be an effective way to reduce the incidence of cancers and thus prevent the perioperative and postoperative anxiety of the patients. Ferrari et al. conducted a comprehensive review on the topic of approaches to the prevention of gynecological malignancies (65). Low body mass index (BMI), women who were obese who reduced their weight, and prolonged use of oral contraceptives, have been found to be associated with a reduction in the incidence of endometrial carcinoma. Using barrier devices (i.e., condoms), human papillomavirus (HPV) vaccination, and abstaining from smoking have been found to reduce the acquisition of HPV infection, thus preventing the development of cervix carcinoma. Annual multimodal screening using the cancer antigen 125 (CA 125) combined with human epididymis protein 4 (HE4) may be an effective biological diagnostic tool for ovarian cancer detection. The above methods aim to prevent the occurrence of gynecologic malignancies or to detect them early to adopt timely and effective therapies, which indirectly reduce the perioperative and postoperative anxiety of the patients.

## Clinical implications

The current review emphasizes the elevated occurrence of perioperative and postoperative anxiety among patients with gynecological cancers (i.e., uterine, cervical, ovarian, endometrial, and vulval cancers). It further suggests that specific nursing interventions (i.e., crisis intervention nursing, multidisciplinary collaborative continuous nursing, psychological nursing, comprehensive psychological nursing, reminiscence therapy involved care, cognitive behavioral stress management, hospital-family integrated continuation nursing, high-quality nursing care, relaxation-focused nursing program, and relaxation/counseling intervention) and psychotropic medications serve as dependable approaches for mitigating perioperative anxiety.

## Study limitations

Nevertheless, it is important to acknowledge certain limitations associated with the interpretation of the findings from the included studies, including small sample sizes, absence of standardized nursing interventions, inconsistent anxiety assessment tools, and the presence of various confounding factors (i.e., age, different types of gynecological cancers, comorbidity, study design, and socioeconomic status). Consequently, there remains a need for a meticulously designed multicenter randomized controlled trial with a substantial sample size and standard nursing interventions to address the perioperative and postoperative anxiety in patients with gynecological cancers.

## Conclusions

To our knowledge, this study is the first to characterize perioperative/postoperative gynecological oncology-associated anxiety. This review highlights that perioperative and postoperative anxiety is a non-negligible clinical issue for patients with gynecological cancers, which should be established in intervention studies.

## Author contributions

BC: Writing – original draft, Software, Investigation. CC: Writing – original draft, Methodology, Investigation. XZ: Writing – review & editing, Formal analysis, Conceptualization. XW: Supervision, Writing – review & editing.
